# Maternal Immune Activation during Pregnancy Alters the Behavior Profile of Female Offspring of Sprague Dawley Rats

**DOI:** 10.1523/ENEURO.0437-18.2019

**Published:** 2019-04-19

**Authors:** Brittney R. Lins, Wendie N. Marks, Nadine K. Zabder, Quentin Greba, John G. Howland

**Affiliations:** Department of Anatomy, Physiology, and Pharmacology, University of Saskatchewan, Saskatoon, SK S7N 5E5, Canada

**Keywords:** behavior, prepulse inhibition, recognition memory, sex differences, sociability

## Abstract

Sex differences are documented in psychiatric and neurological disorders, yet most preclinical animal research has been conducted in males only. There is a need to better understand of the nature of sex differences in brain disease in order to meet the needs of psychiatric patients. We present the behavior profile of adult female offspring produced using a maternal immune activation (MIA) model where pregnant rats receive an immune stimulant and the offspring typically show various abnormalities consistent with psychiatric illnesses such as schizophrenia and autism. The results in female offspring were compared to a previously published cohort of their male siblings (Lins et al., 2018). We examined prepulse inhibition (PPI), sociability, MK-801-induced locomotor activity, crossmodal object recognition (CMOR), and oddity discrimination; behaviors relevant to the positive, negative, and cognitive symptoms of schizophrenia. No between-treatment differences in PPI or locomotor activity were noted. Tactile memory was observed in the control and treated female offspring, visual recognition memory was deficient in the polyinosinic:polycytidylic acid (polyI:C) offspring only, and both groups lacked crossmodal recognition. PolyI:C offspring were impaired in oddity preference and had reduced preference for a stranger conspecific in a sociability assay. Systemic maternal CXCL1, IL-6, and TNF-a levels 3 h after polyI:C treatment were determined, but no relationship was found between these cytokines and the behavior seen in the adult female offspring. Overall, female offspring of polyI:C-treated dams display an array of behavior abnormalities relevant to psychiatric illnesses such as schizophrenia similar to those previously reported in male rats.

## Significance Statement

Sex differences are documented in mental illness and include differences in disease prevalence, symptom presentation, and response to treatment. Despite this, the majority of animal research has been conducted in males only. This study demonstrates the effects of maternal inflammation in pregnancy on long-term behavior outcomes in female offspring, revealing a behavior profile similar to male counterparts. We use a uniquely broad behavior testing battery to show that female offspring from inflammation-exposed pregnancies display an array of abnormal behaviors related to symptom domains of schizophrenia, similar to their male littermates. Maternal cytokine concentrations did not correlate with the severity of these behavior changes suggesting other factors may better indicate long-term disease risk in the offspring.

## Introduction

Adverse events *in utero* and early life are linked to the development of psychiatric illness. Inflammation during pregnancy is associated with increased risk of psychiatric illnesses including autism, schizophrenia, and major depression in the offspring (Patterson, 2011; Jiang et al., 2016; Brown and Meyer, 2018; Gustafsson et al., 2018). The relationship between inflammation and psychopathology is often studied with models of maternal immune activation (MIA) where an immune stimulant such as polyinosinic:polycytidylic acid (polyI:C) is administered to pregnant rodents (Piontkewitz et al., 2012; Brown and Meyer, 2018). Offspring of treated dams display behavioral and neuropathological profiles consistent with psychiatric illness in humans (Brown and Meyer, 2018). The majority of MIA studies focus on the male offspring or lack consideration of sex as a biological variable despite policies by the National Institute of Health and other grant funding agencies that require the examination of sex as a factor in biomedical research (Clayton and Collins, 2014; Coiro and Pollak, 2019). This is particularly concerning for studies of MIA given the sex differences noted in the human psychiatric disorders associated with MIA as a risk factor (Klein and Corwin, 2002; Arad et al., 2017; Brown and Meyer, 2018; Coiro and Pollak, 2019).

Previous studies of the effects of MIA during pregnancy in rats has resulted in extensive knowledge of behavior effects in male offspring. Maternal treatment with polyI:C during gestation results in male offspring with reduced working memory span capacity, dysregulated fear responses, and impaired associative (object-in-place) and crossmodal memory while simple object recognition and object-location memory are largely unaffected, although impaired novel context recognition has also been reported (Wolff and Bilkey, 2010; Wolff et al., 2011; Sangha et al., 2014; Ballendine et al., 2015; Murray et al., 2017). Other studies on adult male offspring from polyI:C-treated pregnancies have shown reduced levels of GAD67 in the dorsal hippocampus which appears to coincide with a loss of hippocampal-frontal coherence and correlate with prepulse inhibition (PPI) deficits (Dickerson et al., 2010, 2013, 2014; Wolff and Bilkey, 2010). PPI has been studied extensively in MIA rat models, yet the results are mixed with several studies showing deficits, no effect, or indicating the timing and type of inflammatory agent determines PPI outcomes (Fortier et al., 2004, 2007; Wolff and Bilkey, 2008, 2010; Ballendine et al., 2015; Hadar et al., 2015). Other studies with offspring of both sexes or sex unspecified report mixed results on PPI as well (Howland et al., 2012; Klein et al., 2013; Van den Eynde et al., 2014; Vorhees et al., 2015). Further MIA studies including male and female rat offspring report altered behaviors such as spontaneous hypolocomotion (Van den Eynde et al., 2014), latent inhibition (Zuckerman and Weiner, 2003, 2005; Zuckerman et al., 2003), and reduced startle (Van den Eynde et al., 2014). Other studies show no change in spontaneous locomotion but reduced sensitivity to the hyperlocomotive effects of amphetamine treatment (Bronson et al., 2011) or hyperlocomotion following amphetamine (Zuckerman et al., 2003; Vorhees et al., 2012) or MK-801 (Zuckerman and Weiner, 2005; but see also Howland et al., 2012). In some tasks, male rat offspring of MIA dams show greater impairments in tasks such as operant conditioning-based set shifting and earlier onset of latent inhibition deficits, reflecting the earlier onset of abnormal developmental trajectories (Piontkewitz et al., 2011a; Zhang et al., 2012; Patrich et al., 2016). While male MIA rats have impaired object-in-place memory, neither control nor MIA females perform this task, possibly reflecting sex-specific differences in baseline task performance (Howland et al., 2012; Ballendine et al., 2015). In a related rat model of MIA, inflammation induced in lactating dams resulted in the development of distinct sex-dependent phenotypes in the suckling offspring, where the females offspring displayed a depressive phenotype and male offspring displayed a psychiatric phenotype (Arad et al., 2017). Taken together, the complicated and often conflicting results from these studies demonstrate the need for sex by treatment analyses in future MIA research (Coiro and Pollak, 2019; Kentner et al., 2019).

The present study aims to contribute to the necessary evaluation of sex effects in the MIA model by highlighting female offspring behavior outcomes in tasks related to the symptoms of schizophrenia and analyzing these results in conjunction with previously published results in their male littermates. The male MIA offspring from the same cohort display hyperlocomotion following MK-801 administration, reduced sociability, impaired visual recognition memory, impaired oddity preference, altered set shifting, and facilitated reversal learning. Using a prospective design, we showed that these behavioral changes did not correlate with acute elevations in a selection of maternal serum cytokines collected 3 h after polyI:C treatment (Lins et al., 2018). We hypothesized that behavior abnormalities would be less severe or absent in early adulthood in accordance with previous literature (Piontkewitz et al., 2011a; Zhang et al., 2012; Patrich et al., 2016). We also correlated behavior of the female offspring with the acute cytokine concentrations from prospectively collected maternal blood and other measurements related to polyI:C treatment to assess relationships between acute maternal cytokine levels, other treatment effects, and offspring behavior, including an oddity discrimination task not previously examined in female rats (Lins et al., 2018).

## Materials and Methods

### Animals

Timed-pregnant Sprague Dawley rats (*n* = 43, Charles River) arrived at the animal holding facility on gestational day 7 (GD7). Primiparous dams were mated between 8 and 10 weeks of age and the presence of a vaginal plug considered GD1. Sires were a minimum of 10 weeks of age at time of mating and their specific breeding history (number of matings, successful pregnancies, mating design, or time between matings) was not guaranteed by the provider. On arrival, pregnant dams were housed individually in standard ventilated (395 × 346 × 227 mm) polypropylene cages. Food (Purina Rat Chow) and water were available ad libitum. The colony room is temperature, but not humidity, controlled (21°C) and maintained on an automatic 12/12 h light/dark cycle with lights on at 7 A.M. Dams were undisturbed until they received treatment on GD15. behavior testing was conducted on adult female offspring (total *n* = 71). All procedures were conducted during the light phase and were conducted in accordance with the Canadian Council on Animal Care guidelines for humane animal use and were approved by the University of Saskatchewan Animal Research Ethics board.

### Maternal treatments and blood samples

**Figure 1.**
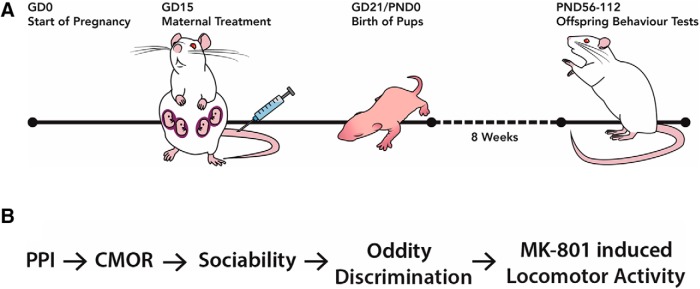
***A***, Schematic detailing the time line of maternal treatment and initiation of offspring behavior testing. Schematic has been published previously (Lins et al., 2018). ***B***, Flowchart depicting the order of the behavior test battery.

Maternal treatment followed previously established protocols in Long-Evans and Sprague Dawley rats (Howland et al., 2012; Zhang et al., 2012; Sangha et al., 2014; Lins et al., 2016, 2018; Paylor et al., 2016; Fig. 1*A*). Additional information is included to improve the scientific rigor of the MIA model as discussed by Kentner et al. (2019). These details also apply to our recent companion publication which tested male rat offspring from the same litters as described here (Lins et al., 2018).

Dam baseline weight (mean = 346.5 g) and rectal temperature were recorded on GD15 immediately before anesthesia with isoflurane (5% induction, 2.5% maintenance, for ∼3 min) to receive a single intravenous tail vein injection of 0.9% saline or polyI:C (4 mg/kg, high molecular weight, InVivoGen; thawed from storage at –20°C). Dams were anesthetized a second time (as above, ∼10 min) 3 h following initial treatment and a blood sample (<1.5 ml total and <6% total blood volume) was collected using a sterile catheter (BD Insyte Autogaurd, 24 GA 0.75 IN 0.7 × 19 mm, REF 381412) from the opposite tail vein used to inject polyI:C or saline. Warm saline was administered once following initial treatment (3 ml), and a second time after blood collection (equal in volume to the collected blood sample). Blood samples coagulated at room temperature for 1 h then centrifuged at 10,000 × *g* for 5 min and serum was stored at –80°C until analysis. ELISAs for CXCL1 (GROα/KC; R&D Systems), CXCL2 (GROβ/MIP-2; R&D Systems), IL-6 (PeproTech), and TNF-α (PeproTech) were performed according to the manufacturer’s instructions and these results are published elsewhere (Lins et al., 2018).

Dams had weight and rectal temperature measured 8, 24, and 48 h after treatment and then were undisturbed for the remainder of their pregnancy. Treatment was administered to *n* = 43 dams, but eight developed hypothermia and were euthanized within 48 h of treatment. Four additional dams experienced body temperature below 36°C but lacked additional indicators of severe sickness or suffering and these were given access to a warming pad on their home cage until their temperature returned to normal (within 24 h). Two dams did not produce viable litters, and two litters had no female offspring. Ultimately, offspring from a total of *n* = 31 litters were included (*n* = 16 polyI:C-treated dams and *n* = 15 saline-treated dams; Table 1). On postnatal day (PND)1, litters were weighed, sexed, and culled to a maximum of 10 (four females where possible). Standard husbandry included cage changes twice per week with one additional cage change during PND14–PND21. Before weaning, all cage changes, feeding, and monitoring of pups was performed by a single investigator to minimize disturbances. On PND23, pups were weaned and housed in same-sex sibling groups of two or three in standard housing as previously described with a PVC tube for enrichment.

**Table 1.** Summary of dams’ treatment, adverse events and litter data

Treatment	Total dams treated	Dams euthanized	Dams w/no litter	Litters included	Viable offspring
Saline	18	0	2 (+1 no ♀)	15	11.94 ± 0.76
PolyI:C	25	8	0 (+1 no ♀)	16	12.00 ± 0.81

Eight dams were killed within 48 h of polyI:C administration because they developed low body temperature and showed sickness behaviors beyond what is acceptable as outlined in our humane intervention protocol. One litter per treatment included male, but no female offspring, resulting in exclusion from the final count of litters included in this manuscript. The two saline-treated control dams that did not produce litters showed no evidence of pregnancy. Viable offspring count per dam includes all surviving offspring of both sexes present on PND1 before culling to a maximum of 10, excludes dams that did not give birth, and is presented as mean ± SEM. All additional data presented on the dams only includes those that produced viable offspring.

### Behavioral testing

Behavior tests were conducted according to published protocols. One or two female offspring per litter were included in each test, except PPI where all available females were included (Table 2). To control for the inherent relationships between siblings, effects from littermates were averaged and one value per litter was used (Zorrilla, 1997; Lazic, 2013). Estrous phase was determined daily between the hours of 7 and 8:30 A.M. before behavior testing. A single investigator used lavage with a p200 pipette and 20 μl of sterile physiologic saline to collect cells from the vaginal wall for immediate visual examination with a light microscope. Proestrus was defined by the presence of uniform nucleated cells, while unnucleated cornified squamous cells were characteristic of estrus, densely packed leukocytes indicated metestrus, and scattered leukocytes alongside nucleated cells indicated diestrus (Hubscher et al., 2005). Estrous determination began 5 d before behavior testing and continued throughout experimentation. All rats displayed a typical 4- to 5-d cycle. Additional handling included exposure to investigators and emphasized picking up and moving the rats until these motions could be conducted with ease, as well as habituation to transport between the housing and testing locations. All animal work occurred during the light phase (7 A.M. to 7 P.M.) with the majority of behavior testing performed between 8:30 A.M. to 5 P.M. Testing began at eight weeks of age (young adulthood) and was completed by 15 weeks of age. The order of testing was PPI, crossmodal object recognition (CMOR), sociability, oddity discrimination, and finally MK-801-induced locomotor activity (Fig. 1*B*). Ethanol (40%) was used to clean all behavior testing equipment between rats.

**Table 2.** Summary detailing the number and litters of female offspring tested in each behavior task

Offspring included in behavior testing												
	Total number		PPI		CMOR		Sociability		Oddity preference		Locomotor activity	
Treatment	Rats	Litters	Rats	Litters	Rats	Litters	Rats	Litters	Rats	Litters	Rats	Litters
Saline	36	15	36	15	22	15	22	15	22	15	14	11
PolyI:C	35	16	35	15	24	15	24	15	24	15	20	13
Number of litters with *n* = 5 or fewer offspring included in PPI												
Treatment	*n* = 5	*n* = 4	*n* = 3	*n* = 2	*n* = 1							
Saline	1	5	1	0	8							
PolyI:C	1	0	7	1	7							
Number of litters with *n* = 1 or *n* = 2 offspring in CMOR, sociability, and oddity												
Treatment	*n* = 2	*n* = 1										
Saline	7	8										
PolyI:C	9	6										
Number of litters with *n* = 1 or *n* = 2 offspring tested in MK-801-induced locomotor activity												
Treatment	*n* = 2	*n* = 1										
Saline	3	8										
PolyI:C	7	6										

All female offspring from *n* = 31 litters completed PPI, while all other tasks included one or two offspring per litter. The unexpected death of one polyI:C rat with no female littermates reduced the number of litters tested to 15. Locomotor activity n was reduced due to some rats being diverted to concurrent research. The number of offspring per litter in each task is further summarized below. behavior scores for littermates were averaged for a single value per litter.

#### PPI

**Figure 2.**
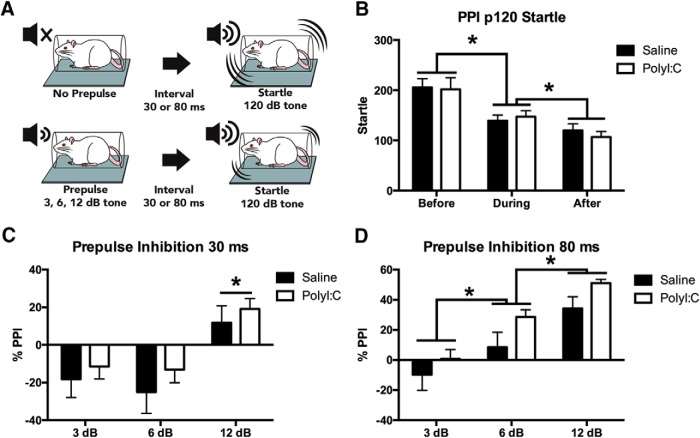
***A***, Schematic illustrating a startle response to a 120 dB tone (top panel) versus the typical reduction in startle reactivity when a prepulse of 3, 6, or 12 dB precedes the startling tone (bottom panel). Schematic has been published previously (Lins et al., 2018). ***B***, Startle reactivity decreased over the course of the PPI testing protocol and polyI:C offspring had significantly higher reactivity at the “after” time point (*p < 0.05). ***C***, There were no differences between groups in %PPI for the short (30 ms) prepulse-pulse interval but %PPI increased with increasing prepulse intensity where the 12 dB prepulse had higher PPI than 2 or 6 dB prepulses. ***D***, There were no differences between groups in %PPI for the long (80 ms) prepulse-pulse interval but %PPI increased with increasing prepulse intensity (3 dB < 6 dB < 12 dB; *p < 0.05).

PPI measures the percentage attenuation of motor response to a startling tone when the tone is preceded by a brief prepulse (Fig. 2*A*; Lins et al., 2018). Two SR-LAb startle boxes (San Diego Instruments) were used. Each session had constant background noise (70 dB) and began with 5 min of acclimatization, followed by six pulse-alone trials (120 dB, 40 ms). Pulse-alone (6), prepulse + pulse (36), and no stimulus (6) trials were then presented in a pseudorandom order, followed by six additional pulse-alone trials. Prepulse + pulse trials began with a 20 ms prepulse of 3, 6, or 12 dB above background (70 dB). Prepulse–pulse intervals (time between the onset of the prepulse and the 120 dB pulse) were short (30 ms) or long (80 ms). The intertrial interval varied randomly from 3 to 14 s (Meyer et al., 2009; Howland et al., 2012; Ballendine et al., 2015).

#### Sociability task

**Figure 3.**
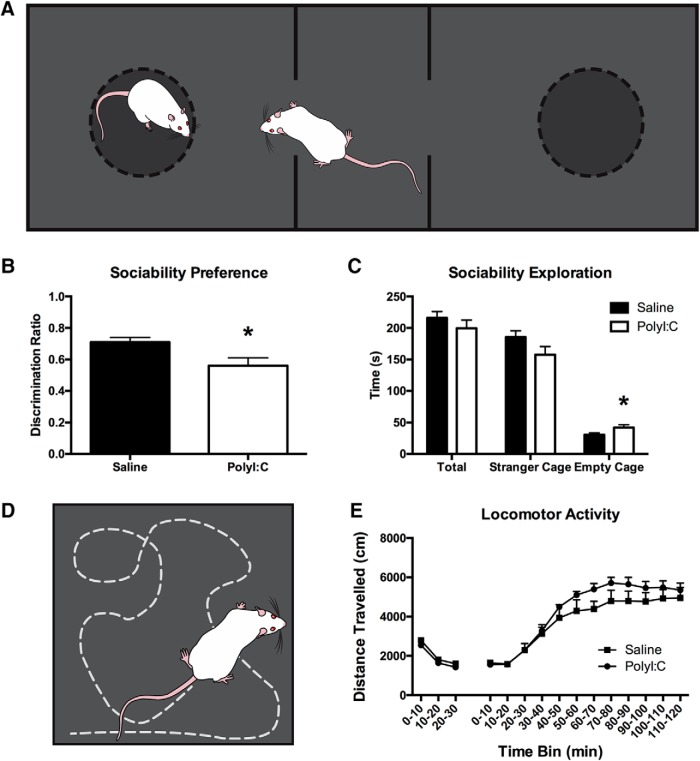
***A***, Schematic representing the black, three-chambered arena used to conduct the sociability task. The chambers on either side of the center start chamber contain identical holding cages, one of which would contain a social stimulus (an age, sex, and treatment matched stranger rat), while the test rat was free to explore. Schematic has been published previously (Henbid et al., 2017; Lins et al., 2018). ***B***, When the exploration data are presented as a DR, both groups show significant preference for the stranger rat; however, polyI:C offspring show significantly less preference when compared to saline offspring. ***C***, There was no significant difference between groups in total exploration or exploration of the social stimulus, although polyI:C rats spent more time exploring the non-social stimulus than saline rats. ***D***, Schematic of the black, square arena where rats’ activity was monitored before and after administration of MK-801. Schematic has been published previously (Lins et al., 2018). ***E***, Graph displaying locomotor activity as distance traveled per 10 min time bin. Both groups had elevated locomotor activity following MK-801 administration, but there was no effect of maternal treatment. *p < 0.05.

The testing apparatus was a rectangular arena (150 × 40 cm) of black corrugated plastic divided into three compartments, one middle start compartment (30 × 40 cm) and two ‘stranger’ compartments on either side (60 × 40 cm, see Fig. 3*A*; Henbid et al., 2017; Lins et al., 2018). The walls dividing the middle compartment from the stranger compartments were clear Plexiglas (extend 12 cm from each wall leaving a 16-cm opening allowing travel between compartments) and removable black opaque barriers which, when inserted, prevented entry into the stranger compartments. Each stranger compartment contained a circular mesh cage (18 cm in diameter, 20 cm in height) with hinged lid (3/4” plywood, painted matte black). The height of the cage was extended 20 cm with vertical metal rods to discourage climbing. The task began with 10 min habituation with the barriers removed. The test rat was then contained in the middle section with the barriers in place and a stranger rat was placed in one of the mesh cages. The barriers were removed, and the test rat explored for an additional 10 min. Video recording and locomotor activity tracking was done with EthoVision software, and videos were manually scored with a stopwatch by a trained investigator blind to treatment status, and the opaque cage roof obscured the location of the stranger rat. Stimulus exploration was scored when the test rat directly approached (watching, contacting, sniffing, or circling) each of the cages, with the face of the rat oriented toward the cage at a maximum distance of 2 cm. All stranger rats were sex, age, and treatment matched to the test rat (Bitanihirwe et al., 2010; Henbid et al., 2017).

#### MK-801-induced locomotor activity

The apparatus was a square arena (40 × 40 × 60 cm) made of black corrugated plastic (Fig. 3*D*; Lins et al., 2018). A camera mounted to the ceiling recorded all activity and EthoVision software was used to track activity. Rats were tested four at a time, with each rat placed in one of four separate arenas for 30 min of habituation. Immediately following, rats were administered MK-801 (0.1 mg/kg, i.p.) and placed back into the arena for an additional 120 min. Activity was recoded with Noldus EthoVision XT 11.5 software.

#### Visual, tactile, and CMOR

**Figure 4.**
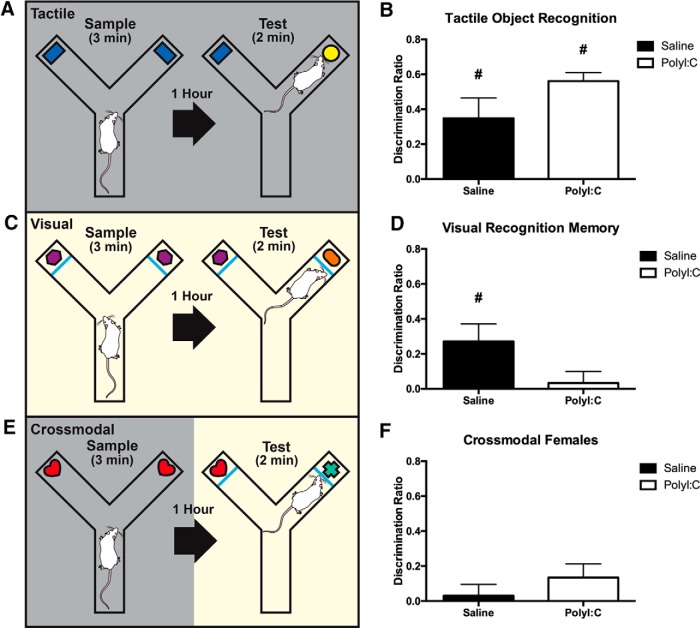
***A***, The Y-maze assembled to conduct the tactile phase in red light conditions where the rat is able to explore objects via touch. ***B***, both groups of offspring display robust novelty preference in the tactile phase with novel object exploration significantly greater than chance levels. ***C***, The Y-maze assembled for the visual phase which is conducted in white light conditions with the addition of a clear, Plexiglas window to prevent tactile exploration of the objects, limiting the rats to visual observation. ***D***, Saline offspring demonstrated visual memory with novel object exploration significantly greater than chance but polyI:C offspring did not perform above chance levels. ***E***, The Y-maze assembled for the crossmodal phase which has a tactile sample phase and visual test phase. ***F***, both groups failed to display crossmodal recognition memory as novel object exploration was equal to chance; # indicates significant difference from chance exploration (DR = 0, *p* < 0.05) in a single sample *t* test. Schematic has been published previously (Lins et al., 2018; Paylor et al., 2018).

This task uses spontaneous exploratory behavior to assess visual memory, tactile memory, and visual-tactile sensory integration (Winters and Reid, 2010; Jacklin et al., 2012). The testing apparatus was a Y-shaped maze with one start arm and two object arms (10 × 27 cm) made of white corrugated plastic (Fig. 4; Lins et al., 2018; Paylor et al., 2018). A white plastic guillotine-style door separated the start arm from the object arms, and Velcro at the distal end of the object arms fixed objects in place. A removable, clear Plexiglas barrier could be inserted in front of the objects. A tripod positioned above the apparatus held a video camera that recorded the task activity. Rats were habituated to the apparatus twice for 10 min. Lighting alternated during habituation between white light (during visual phases) and red light (during tactile phases) for 5 min each with the order counterbalanced, and the clear barriers were in place for 1 d of habituation and removed for the other with order counterbalanced. Test days consisted of a 3 min sample phase with two identical copies of an object attached with Velcro to the maze, a 60-min delay, and then a 2-min test phase with a third copy of the original object and a novel object placed in the maze. Rats began each phase in the start arm; the guillotine door was opened and closed once the rat entered the object arms. This task consisted of three distinct tests performed on three separate days in the following sequence: tactile memory (day 1; Fig. 4*A*), visual memory (day 2; Fig. 4*C*) and crossmodal memory (day 3; Fig. 4*E*). Red light illuminated the tactile phases allowing the rats’ behavior to be recorded while preventing the rats’ visual assessment of the objects and the removal of the clear barriers allowed for tactile exploration. White light was used during visual phases, but clear Plexiglas barriers in front of the objects prevented tactile exploration. CMOR had a tactile sample phase (red light, no barriers) and a visual test phase (white light, clear barriers). Recognition memory was defined as significantly greater exploration of the novel object than the familiar object. Behavior recordings were manually scored with a stopwatch by investigators blind to the treatment status of the rats and identity of the objects (Winters and Reid, 2010; Ballendine et al., 2015).

#### Oddity discrimination

**Figure 5.**
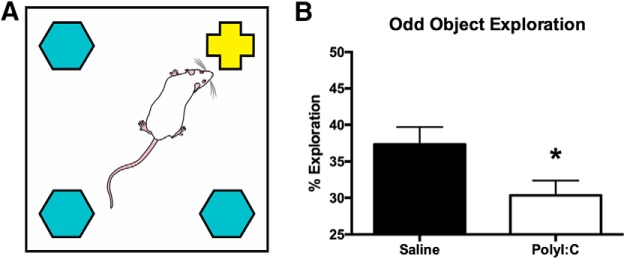
***A***, Schematic of the white square arena used to conduct the oddity discrimination task showing the arrangement of three identical objects and one different, or odd object. Schematic has been published previously (Lins et al., 2018). ***B***, bar graph displaying the percentage of total object exploration spent examining the odd object. PolyI:C offspring displayed significantly less oddity preference than saline offspring (*p < 0.05).

The testing apparatus was a square arena (60 × 60 × 60 cm) constructed of white corrugated plastic with Velcro in each of the four corners. Two days of habituation to the arena (10 min sessions) preceded the test day. On test day, three identical objects made of glass or plastic and one different, or “odd” object were fixed to the Velcro locations (Fig. 5*A*; Lins et al., 2018) and the rats’ activity were recorded for 5 min using a video camera mounted to the ceiling. Object exploration times were manually scored using a stopwatch by an investigator blind to the treatment status of the rats (Bartko et al., 2007a). Object examination was counted when a rat’s face was oriented toward the object at a maximum distance of 2 cm.

### Statistical analyses

A between-subjects design was used, and analyses were conducted with independent samples *t* tests, one sample *t* tests, and ANOVAs using Statistical Package for the Social Science version 22 (IBM). Outliers were defined as having a performance metric falling >2 SDs from the mean and were removed from analysis on a case by case basis. Outliers were identified and removed before calculating litter averages to prevent excessive exclusion of data points. One saline and two polyI:C rats were removed from visual recognition memory, but all litters remained represented. Two polyI:C litters were excluded from analysis for startle to the P120 tone, one polyI:C litter was removed from the analysis for PPI 30 ms interval and PPI 80 ms interval. No litters or individuals were removed from the Oddity, Sociability, or Locomotor task analyses. Estrous phase was incorporated into analysis as a covariate, however no consistent patterns were observed, potentially due to a low n of rats in each phase. Sphericity violations were accounted for using the Greenhouse–Geisser adjustment and degrees of freedom were adjusted when Levene’s Test was violated. The use of one-tailed and two-tailed tests is specified for each task. Relationships between maternal serum cytokine concentrations and long-term offspring outcomes were determined using bivariate correlations followed by a Benjamini–Hochburg (B–H) adjustment to control for multiple comparisons (Benjamini and Hochberg, 1995). All data are presented as group mean ± SEM, and asterisks indicate a significant difference between groups with a 95% confidence interval (*p* < 0.05). The pound symbol (#) is used to indicate a significant difference from a chance result.

## Results

The acute effects of saline and polyI:C treatment on this cohort of pregnant dams and neonatal pups have been published previously (Lins et al., 2018). Briefly, dams treated with polyI:C had reduced body weight compared to saline when followed up at 8, 24, and 48 h after treatment, but no significant change in body temperature at the same timepoints. Maternal serum collected 3 h after treatment was analyzed with ELISA for concentrations of cytokines CXCL1, CXCL2, IL-6, and TNF-α. CXCL1 and IL-6 were significantly elevated in polyI:C-treated dams. On PND1, the average offspring mass (males and females pooled) from polyI:C-treated litters was significantly less than controls but there was no difference in litter size between the groups (Lins et al., 2018).

### Maternal polyI:C treatment failed to significantly affect startle or PPI

Startle responses to acoustic stimuli were assessed by measuring startle alone and PPI in saline (*n* = 15 litters) and polyI:C female offspring (*n* = 15 litters). Startle to the 120 dB pulses alone decreased during the session (main effect of time: *F*_(1.35,36.45)_ = 26.26, *p* < 0.001; Fig. 2*B*) but no treatment or interaction was present. For prepulse trials with a 30 ms (short) interval, a main effect of prepulse intensity on PPI (*F*_(2,56)_ = 40.33, *p* < 0.001; Fig. 2*C*) was found with no effect of treatment. Overall, PPI was greater at 12 dB compared to 3 and 6 dB (*p* < 0.001). For trials with an 80 ms (long) prepulse-pulse interval, a main effect for prepulse intensity was found (*F*_(2,56)_ = 89.37, *p* < 0.001; Fig. 2*D*) for PPI, but no effect of treatment and no interaction. Overall, PPI increased with louder prepulses.

### PolyI:C offspring have sociability deficits

Both groups of female offspring (*n* = 15 saline litters, *n* = 15 polyI:C litters) displayed a significant preference for the cage containing an unfamiliar rat compared to the empty cage when analyzed using a within-subjects design (saline: *t*_(14)_ = 14.18, *p* < 0.001; polyI:C: *t*_(14)_ = 8.11, *p* < 0.001). When the results were compared between treatment groups, female polyI:C rats had a significantly lower discrimination ratio (DR; calculated as exploration_stranger_ – exploration_empty_/exploration_total_; *t*_(28)_ = 2.61, *p* < 0.05; Fig. 3*B*) and spent significantly more time exploring the empty cage compared to the saline controls (*t*_(28)_ = –2.59, *p* < 0.05; Fig. 3*C*). There was no difference in total exploration times (*p* > 0.05).

### Both groups of offspring increase locomotor activity following MK-801 administration

Locomotor data comparing female polyI:C (*n* = 13 litters) and saline (*n* = 11 litters) offspring were analyzed with a mixed repeated measures ANOVA (Fig. 3*E*). Results revealed a main effect of Time (*F*_(2.47,62.24)_ = 62.24, *p* < 0.001; Fig. 3*E*) but no treatment effect and both groups displayed increased locomotion after MK-801 administration.

### PolyI:C offspring perform tactile, but not visual, object recognition memory and neither group display crossmodal recognition

All CMOR data are presented as a DR (exploration_novel_ – exploration_familiar_/exploration_total_) for the first minute of the test phase. One-tailed single sample *t* tests compared each group’s exploration to chance (DR of 0). Both groups demonstrated significant tactile object recognition memory (saline: *t*_(14)_ = 3.00, *p* < 0.01; polyI:C *t*_(14)_ = 11.53, *p* < 0.001; Fig. 4*B*). PolyI:C females did not perform above chance for visual memory (polyI:C: *t*_(13)_ = 0.49, *p* > 0.05), although saline offspring showed significant preference for the novel object (*t*_(15)_ = 2.72, *p* < 0.05). In the crossmodal phase, both groups of female rats failed to show a preference for the novel object (saline females: *t*_(14)_ = 0.46, *p* > 0.05; polyI:C females: *t*_(14)_ = 1.71, *p* > 0.05). There were no differences in total object exploration times between groups in any of the sample and test phases (Table 3).

**Table 3.** Exploration times (s) for each phase of the CMOR task, presented as μ ± SEM

Treatment	Task phase	Tactile	Visual	Crossmodal
Saline	Sample	61.10 ± 2.49	6.41 ± 0.56	66.21 ± 4.73
	Test	36.71 ± 2.41	3.50 ± 0.61	3.36 ± 0.36
PolyI:C	Sample	60.32 ± 3.07	7.18 ± 0.83	58.61 ± 4.43
	Test	34.57 ± 3.42	2.72 ± 0.28	3.84 ± 0.27

### PolyI:C-treated offspring have reduced oddity preference compared to saline offspring

Saline (*n* = 15 litters) and polyI:C (*n* = 14 litters) offspring both explored the odd object at a greater than chance level (saline: *t*_(14)_ = 5.27, *p* < 0.001; polyI:C: *t*_(13)_ = 2.66, *p* < 0.05; Fig. 5*B*) when analyzed with a single sample *t* test against a value of 25%. When the groups were compared directly, saline offspring spent a significantly greater % exploration with the odd object compared to polyI:C offspring (*t*_(27)_ = 2.24, *p* < 0.05; Fig. 5*B*). There was no difference in total exploration time between saline (97.53 ± 4.08 s) and polyI:C rats (98.94 ± 4.79 s, *t*_(27)_ = –0.23, *p* < 0.05).

### Correlations between measurements taken of the dams during pregnancy and offspring outcomes

Measurements taken during pregnancy were correlated with long-term behavior outcomes in the female offspring. Serum cytokine concentrations of CXCL1, CXCL2, IL-6 and TNFα were determined from blood samples collected from the dams 3 h after treatment and analyzed with ELISA. Additional effects of treatment were determined through monitoring with weight and rectal temperature measurements taken 8, 24, and 48 h after treatment (Lins et al., 2018). These data were correlated to behavior of the offspring using bivariate correlations. Maternal weight changes following treatment (anesthesia with saline or polyI:C administration and blood sampling) was the only variable associated with offspring behavior. Greater weight loss in polyIC-treated dams 8 h after treatment was associated with reduced startle response in their female offspring during the initial tone-alone trials (*r* = 0.623, *p* < 0.05, B–H *p* < 0.05), and this was not seen in the saline group (*r* = 0.44, *p* > 0.05). Weight loss in the saline dams at 24 h after treatment was correlated to lower %PPI at the 80 ms interval (*r* = –0.555, *p* < 0.05, B–H *p* < 0.05). Saline dam weight loss 8 h after treatment was related to the DR in the sociability task (*r* = 0.716, *p* < 0.01, B–H *p* < 0.05). The importance of these relationships is difficult to gauge due to no significant effects of saline treatment on weight, and the lack of treatment effects in startle or PPI. Previous studies have also shown mixed results regarding maternal weight changes and offspring behavior outcomes (Wolff and Bilkey, 2010; Vorhees et al., 2012). Dedicated studies will be necessary to determine the reliability and potential importance of these results.

### Male and female offspring show similar behavioral profiles in response to MIA

The data presented in this paper were further analyzed in conjunction with the male littermates from Lins et al. (2018) with sex and treatment as factors using 2 × 2 factorial ANOVAs. It should be noted that this analysis necessitates including two values per litter (for each sex) which violates the assumption that subjects are independent because littermates are inherently related. No main effect of treatment (*F*_(1,60)_ = 0.59, *p* > 0.05) or sex (*F*_(1,60)_ = 0.25, *p* > 0.05), and no sex by treatment interaction (*F*_(1,60)_ = 0.11, *p* > 0.05) was found for 30 ms interval PPI. For 80 ms interval PPI trials, no main effect of treatment (*F*_(1,56)_ = 0.10, *p* > 0.05) or sex (*F*_(1,56)_ = 0.82, *p* > 0.05) was found, but a sex by treatment interaction was shown (*F*_(1,56)_ = 4.07, *p* < 0.05). A Tukey HSD *post hoc* test revealed saline females had lower PPI than saline males (*p* < 0.05). Main effects of treatment (polyI:C offspring have a lower DR than saline offspring; *F*_(1,58)_ = 9.55, *p* < 0.01) and sex (female offspring have a lower DR than male offspring; *F*_(1,58)_ = 4.87, *p* < 0.05) were found for sociability, with no significant interaction (*F*_(1,58)_ = 0.13, *p* > 0.05). Tactile object recognition memory did not differ by treatment (*F*_(1,59)_ = 0.30, *p* > 0.05) or sex (*F*_(1,59)_ = 1.06, *p* > 0.05); however, but a significant sex by treatment interaction was revealed (*F*_(1,59)_ = 4.25, *p* < 0.05). Tukey HSD *post hoc* testing failed to reveal any significant differences between individual groups. Visual object recognition memory was not affected by treatment (*F*_(1,56)_ = 3.60, *p* > 0.05) or sex (*F*_(1,56)_ = 0.21, *p* > 0.05), and no interaction was present (*F*_(1,55)_ = 0.003, *p* > 0.05). CMOR memory was not affected by treatment (*F*_(1,58)_ = 0.17, *p* > 0.05) or sex (*F*_(1,58)_ = 0.01, *p* > 0.05) and there was no interaction between these factors (*F*_(1,58)_ = 3.98, *p* > 0.05). A main effect of treatment (*F*_(1,55)_ = 19.30, *p* < 0.001, polyI:C offspring explore the odd object less than saline offspring) was found for oddity, in the absence of a main effect of sex (*F*_(1,55)_ = 0.03, *p* > 0.05) or sex by treatment interaction (*F*_(1,55)_ = 1.88, *p* > 0.05). Locomotor activity was not analyzed for sex by treatment interactions due to known differences in MK-801 metabolism and the use of different doses in males and females (Andiné et al., 1999).

## Discussion

The adult female offspring of rat dams that received an immune stimulant during pregnancy displayed various behavior abnormalities compared to the offspring of saline-treated dams. The polyI:C-treated offspring had reduced sociability, impaired visual discrimination, and lack of preference for an odd object compared to offspring from control litters. both treatment groups displayed heightened locomotor activity in response to MK-801 administration and tactile recognition memory was intact in both groups. Neither group of offspring demonstrated crossmodal memory, and there were no treatment effects on PPI. These results complement a companion paper that assessed the male offspring (Lins et al., 2018), and by directly analyzing sex by treatment interactions where possible, we shown that MIA during pregnancy had similar effects on both sexes of offspring.

A significant limitation of this study is the use of timed-pregnant dams. Several studies show an impact of travel stress on the dams and offspring. For example, Moriyama et al. (2013) examined the effect of transport stress on seizure susceptibility in the offspring and found an increase in variability in those transported during gestation; however, maternal care behavior had a greater impact than transport stress on seizure susceptibility. We have previously reported no observed changes to maternal behavior following polyI:C administration (Zhang et al., 2012), but we did not assess this directly in this cohort or strain. Shipment stress also increases susceptibility to the valproate-induced developmental toxicity model of autism (Ogawa et al., 2007; Kuwagata et al., 2009). We are unable to confirm if shipment stress had a similar impact in our study. Despite these limitations, many comparable studies on development and gestational adverse events have relied on the use of timed-pregnant dams (Lodge and Grace, 2001; Du and Grace, 2013, 2016a,b; Van den Eynde et al., 2014; Ballendine et al., 2015; Lins et al., 2018). Recently, Kentner et al. (2019) highlighted that consideration of all MIA protocols will enable comprehensive understanding of their impacts on offspring outcomes. Thus, we believe our results are of value.

### Lack of sex-specific effects of polyI:C treatment on behavior of the offspring

We previously reported that male polyI:C offspring from this cohort displayed greater startle to the 120 dB tone at the end of the PPI protocol compared to saline males. Although the effect in the males was small and limited to a single parameter, the female data presented here shows no effect of MIA on any measure of PPI and acoustic startle response. The effects of MIA on PPI in rodent models are mixed with many studies showing PPI impairments in the offspring of immune challenged rats (Borrell et al., 2002; Romero et al., 2007; Wolff and Bilkey, 2008, 2010; Dickerson et al., 2010, 2013, 2014; Howland et al., 2012; Klein et al., 2013; Ballendine et al., 2015; Hadar et al., 2015) and mice (Ozawa et al., 2006; Smith et al., 2007), including impairments seen in both sexes (Meyer et al., 2009; Howland et al., 2012; Basta-Kaim et al., 2015). Other studies show no effects of MIA on PPI, similar to our observations (Missault et al., 2014; Van den Eynde et al., 2014; Vorhees et al., 2015; Lins et al., 2018). Sex effects in PPI in general have been reviewed and the influence of female sex hormone fluctuations have been studied (Kumari, 2011). High estrogen phases of the menstrual cycle have been associated with lower PPI, although this is not consistently observed (Swerdlow et al., 1997; Jovanovic et al., 2004; Kumari et al., 2008). Additionally, PPI disruption by a 5HT_1A_ agonist can be prevented by administration of exogenous estrogen and progesterone in rats which may imply a protective role of sex hormones against PPI disruption (Gogos and Van den Buuse, 2004). The results from the present study do not support the claim of a strong influence of estrous phase on PPI performance; however, it should be noted that estrus was not controlled for and the ability of the present study to detect an effect may be underpowered.

In the sociability task, polyI:C-treated male offspring spent less time exploring a same-sex, unfamiliar conspecific compared to saline controls (Lins et al., 2018). We observed a different pattern of reduced sociability in the female polyI:C offspring, indicated by a significantly lower DR compared to the saline offspring which was driven by significantly more time exploring the empty cage on the opposite side of the apparatus. The social exploration data are presented as a comparison between the saline and polyI:C offspring (comparing the relative degree of social preference), while others have presented the data as a within-subjects comparison to report either the presence or absence of social preference (Silverman et al., 2010). We believe comparing treatment groups allows the detection of subtle behavior differences that could be missed in instances where stimuli with a substantial difference in salience (such as an unfamiliar rat vs an empty cage) result in high DRs, and this natural preference would need to be abolished to show a treatment effect. Natural preference for social stimuli versus objects is documented in rodents (Lee and Green, 2016). Presenting the data as a between groups comparison also allows direct evaluation alongside previously published sociability data from our lab, including that of the male littermates from this cohort (Henbid et al., 2017; Lins et al., 2018). The use of single sample comparisons may be best suited to tasks that are challenging for control animals to complete, such as the complex visual discrimination tasks including visual and crossmodal recognition memory where visual stimuli are less salient and the resulting DRs tend to be lower (Winters and Reid, 2010). Both strategies of data analysis are common in behavior literature, and factors such as strength or salience of stimuli and task difficulty should be considered when representing data. Overall, both male and female polyI:C offspring display a deficit in sociability compared to controls, but this presents in a subtly different manner depending on sex and may be related to previously observed differences in PFC development (Piontkewitz et al., 2011b; Paylor et al., 2016), The direct significance of MIA-induced developmental trajectory differences to the aberrant social behavior observed here remains to be determined.

The effects of MK-801 administration on locomotor activity have been reported in previous studies with mixed results (Zuckerman and Weiner, 2005; Howland et al., 2012; Vorhees et al., 2012; Missault et al., 2014). The male siblings in this cohort were significantly affected by a dose of 0.2 mg/kg IP indicated by heightened locomotor activity which was not seen in the control males (Lins et al., 2018). The females in this paper were given a lower dose of 0.1 mg/kg comparable to other studies (Andiné et al., 1999; Howland et al., 2012; Zhao et al., 2013). Unfortunately, both the saline and polyI:C females showed increased locomotion which confounds the ability to discern whether prenatal polyI:C treatment affected sensitivity to MK-801.

In the CMOR task, both saline and polyI:C females demonstrated object recognition in the tactile phase of the test, similar to what was seen in males of the same cohort (Lins et al., 2018) and Long Evans males (Ballendine et al., 2015). PolyI:C-treated offspring were impaired in the visual phase, and neither group performed significantly different from chance exploration in the crossmodal phase, a notable distinction from the crossmodal memory exhibited by saline-treated males (Lins et al., 2018). Previous studies on MIA offspring have shown reduced discrimination in object memory behavior tasks in females and lower DRs are common in the crossmodal task, possibly reflecting task difficulty (Howland et al., 2012; Ballendine et al., 2015; Marks et al., 2016). Visual and crossmodal memory depend on the perirhinal cortex while the posterior parietal cortex is necessary for tactile memory, suggesting there may be regionally specific deficits as a result of MIA (Winters and Reid, 2010; Jacklin et al., 2016).

Oddity preference and perception have been assessed in several tasks using rats and mice (Bussey et al., 2005; Bartko et al., 2007a,b; Cowell et al., 2010; Cloke et al., 2016; Marks et al., 2018; Paylor et al., 2018); however, to our knowledge this is the first study to assess this oddity task in female rats. Improved understanding of the nature of oddity discrimination is relevant for the successful management of cognitive impairment in conditions such as schizophrenia, a symptom domain highly related to patient functional outcomes (Cloke et al., 2016). Prenatal polyI:C treatment affected females in the same manner as males with a significant reduction in oddity preference compared to saline offspring (Lins et al., 2018). The successful performance of oddity preference depends on multisensory integration similar to CMOR, yet distinct in that visual and tactile associations can be formed simultaneously and there is no mnemonic demand in the oddity task (Cloke et al., 2016). Multisensory integration is disrupted by NMDA receptor antagonism using ketamine in the orbitofrontal cortex and reversed with α_4_β_2_ nicotinic acetylcholine in a GABA_A_-dependent mechanism (Cloke et al., 2016) and abnormalities in these brain regions and receptor types may be good candidates to explore in future studies of MIA and oddity preference.

The degree to which these behavioral effects replicate or contradict previous data varies. The MIA literature displays a lack of reproducibility, which may be due to variety of protocols used. Procedural variations in model species and strain, timing of inflammatory insult, inflammatory agent, and dose and route of administration have the potential to alter experimental outcomes. Other details such as rodent housing (bedding type, pathogen-free status, temperature, etc.), parental age, maternal experience, food and water quality, cage companions, and age at weaning, which are not commonly reported, may influence outcomes and reduce reproducibility of the model (Smolders et al., 2018; Kentner et al., 2019). The basic protocol used here is relatively common in MIA literature, yet very similar protocols yield contrasting behavior responses; for example, hypolocomotion versus hyperlocomotion in an open field (Van den Eynde et al., 2014; Lins et al., 2018). Comparison in this case is complicated by the use of spontaneous versus drug-enhanced locomotor paradigms and the collection of maternal blood samples (Van den Eynde et al., 2014; Lins et al., 2018). Indeed, distinct neuopathological alterations were noted with microglia activation in MIA offspring found in one study (Van den Eynde et al., 2014), while previous work from our group has found no changes in microglia in offspring generated in our laboratory (Paylor et al., 2016). These results support the notion that enhanced reporting of such variables is warranted, and dedicated future studies should assess the effects of such procedural differences directly (Goldstein et al., 2014; Careaga et al., 2018; Kentner et al., 2019; Mac Giollabhui et al., 2019).

### Implications for sex differences in the MIA model

MIA caused by polyI:C administration resulted in altered behavior in female offspring in multiple behavior tasks including sociability, visual and crossmodal memory, and oddity preference. Overall, the present data do not provide strong evidence for sex differences in response to polyI:C treatment. Inflammation in pregnancy relates to the etiology of sexually dimorphic disorders, notably schizophrenia and autism (Davis and Pfaff, 2014; Goldstein et al., 2014; Patel et al., 2018). The lack of overt sex differences observed in the present study suggest the MIA model in rats may be limited in this respect, although others have shown more promising results in this regard (Piontkewitz et al., 2011a; Zhang et al., 2012).

**Table 4.** Summary of the effects of MIA on female offspring alongside the male offspring from the same cohort in a previously published companion paper (Lins et al., 2018**)**

Behavior test	Males	Females
PPI	-	-
MK-801 locomotion	↑ 47.72%	n.d.
Sociability	↓ 22.97% (n.s.)	↓ 21.13%
Tactile memory	-	-
Visual memory	↓ 90.33%	↓ 87.87%
Crossmodal memory	↓ 93.71%	-
Oddity preference	↓ 31.57%	↓ 18.69%

↑, heightened response or facilitation; ↓, diminished response or impaired performance in comparison to a control group; –, no significant change compared to controls; n.d., not determined. Percentage change was calculated as a comparison to the equivalent control group [(saline – polyI:C/saline) × 100]. Male locomotor data were calculated from the total distance traveled after MK-801 administration. For consistency, both male and female sociability percentage change was calculated using the DR data, although it should be noted the DR was a non-significant (n.s.) effect in the males and they instead spent significantly less time (s) exploring the social stimulus than controls.

## Synthesis

Reviewing Editor: Quentin Pittman, University of Calgary Decisions are customarily a result of the Reviewing Editor and the peer reviewers coming together and discussing their recommendations until a consensus is reached. When revisions are invited, a fact-based synthesis statement explaining their decision and outlining what is needed to prepare a revision will be listed below. The following reviewer(s) agreed to reveal their identity: Hanna Stevens.

The manuscript “Maternal immune activation during pregnancy alters the behavior profile of female offspring in Sprague-Dawley rats” provides an important contribution to our understanding of prenatal immune challenge models. The focus on female offspring is important and provides a test bed to further explore sex differences in the MIA model. To a certain extent it is unfortunate that the authors chose not to publish these data with those previously obtained from male offspring. Nonetheless, the authors demonstrate that adult female offspring of rat dams that received an immune stimulant during pregnancy display behavior abnormalities, including reduced sociability, impaired visual discrimination, and lack of preference for an odd object compared to offspring from control litters.

In general, the experiments are well-designed, the findings are robust and appropriately interpreted, and the manuscript is clear and well-written. Nonetheless there are some major items and several more minor ones that require attention in revision.

Major:

1. The most significant concern is that very little is written or considered for why this manuscript has results that are similar or different than previous findings in the field. Some component of these results here are unique, but several have been done repeatedly, even examining sex. The authors should give substantially more attention to justifying this experiment, which should be based on thoroughly examining protocol differences between this and previous studies and understanding what similarities or differences play a role in outcomes. They should discuss the timing, dosage, and route of these different studies as these are critical for the neurodevelopmental processes that are being targeted by MIA. Behavioral outcomes of the MIA model can be influenced by a number of experimental design consideration (see Kentner et al 2018). The current summary of sex differences across MIA model studies does not acknowledge this challenge of comparing outcomes across laboratories.

2. A major concern is that the animals were shipped during gestation. This should be clearly raised as a major limitation of the study in the discussion. There are papers that address this ( eg Moriyami et al (2013) Epilepsy Behav29:19-27) and these and others should be cited and highlighted in the discussion.

Minor:

1. Introduction: here address the issue # 1 above. Also many of the MIA model review references could be updated with more recent citations.

2. Methods: (line 136, Page 7) - Please summarize the maternal outcome data (number of dams treated, euthanized, viable litter numbers etc.) in a table.

3. Methods: Methods (line 142, Page 7) - Ultimately, offspring from a total of n=32 litters were included (n=16 polyI:C treated dams and n=16 saline treated dams). How many pups per litter? It would be helpful to see this information in a table.

4. Methods: The authors describe that 1-2 females from each of 32 litters were used. The maximum number given these latter descriptions is 64 but the authors state that 71 offspring were behaviorally tested. Can the authors please clarify the correct sample sizes?

5. For dams that were excluded, please describe their group membership so that the control and experimental treatments can be assessed for their adverse outcomes.

6. Statistical Analysis (line 242, Page 12) - How many outliers were removed?

7. Given the desire to examine sex differences, the authors should quantify the extent (perhaps percent change) of differences in table 2 so that this kind of comparison could be made. Direct comparisons of the data showing main effects of sex and/or interactions should also be displayed in a table.

8. Given the focus on sex differences, the authors should address the weight differences at P1 measured and displayed in Lins et al 2018, but in this manuscript, depict those female offspring

9. The schematic images in all the figures appear to have been published before without any acknowledgment of this

10. For the social task, the authors describe coding by hand ‘interaction’ but then discuss in the results ‘exploration.’ Are these the same? If they are and both are used for both of the data types displayed in the figure, the social coefficients displayed seem unlikely given the w ay the authors describe calculating them and given the total times displayed. Furthermore, the measures of sociability generated in the three-chamber assay have often been interpreted as an all-or-none result that should be restricted to comparison with a single experimental group, rather than a comparison between groups (Silverman et al., 2010). The rationale for comparing the groups could be integrated in the discussion.

11. Discussion: the authors play down the significant correlations they find between pregnancy variables and outcomes in the saline groups. For the reader to be able to judge the significance of these themselves, correlations should be graphically displayed, showing both the saline and MIA groups. IN fact, if there are correlations in the saline group that ‘break down’ in the setting of MIA, this could be seen in a straightforward manner graphically and discussed if observed to be distinctly different.

12. Discussion: (line 441, Page21) - “Maternal CXCL1, IL-6, and TNF-a concentrations are not of value in predicting these abnormalities, therefore inquiry into other factors may provide a stronger indication of long-term disease risk in the adult offspring.” Given that maternal samples were only collected at a single time point (3hr post injection), it is possible that the peak of maternal cytokine response could have occurred later (especially for IL6 - see Careaga et al 2018). The final statement should be reworded.

13. The authors do little to discuss how the absence or presence of sex differences is relavant to psychiatric illness that have or don't have significant sex differences in their rates. This should be appropriately considered.

## References

[b1] Andiné P, Widermark N, Axelsson R, Nyberg G, Olofsson U, Mårtensson E, Sandberg M (1999) Characterization of MK-801-induced behavior as a putative rat model of psychosis. J Pharmacol Exp Ther 290:1393–1408. 10454519

[b2] Arad M, Piontkewitz Y, Albelda N, Shaashua L, Weiner I (2017) Immune activation in lactating dams alters sucklings’ brain cytokines and produces non-overlapping behavioral deficits in adult female and male offspring: a novel neurodevelopmental model of sex-specific psychopathology. Brain Behav Immun 63:35–49. 10.1016/j.bbi.2017.01.015 28189716

[b3] Ballendine SA, Greba Q, Dawicki W, Zhang X, Gordon JR, Howland JG (2015) behavioral alterations in rat offspring following maternal immune activation and ELR-CXC chemokine receptor antagonism during pregnancy: implications for neurodevelopmental psychiatric disorders. Prog Neuropsychopharmacol Biol Psychiatry 57:155–165. 10.1016/j.pnpbp.2014.11.002 25445065 PMC4464825

[b4] Bartko SJ, Winters BD, Cowell RA, Saksida LM, Bussey TJ (2007a) Perceptual functions of perirhinal cortex in rats: zero-delay object recognition and simultaneous oddity discriminations. J Neurosci 27:2548–2559. 10.1523/JNEUROSCI.5171-06.2007 17344392 PMC6672512

[b5] Bartko SJ, Winters BD, Cowell RA, Saksida LM, Bussey TJ (2007b) Perirhinal cortex resolves feature ambiguity in configural object recognition and perceptual oddity tasks. Learn Mem 14:821–832. 10.1101/lm.749207 18086825 PMC2151019

[b6] Basta-Kaim A, Fijał K, Ślusarczyk J, Trojan E, Głombik K, Budziszewska B, Leśkiewicz M, Regulska M, Kubera M, Lasoń W, Wędzony K (2015) Prenatal administration of lipopolysaccharide induces sex-dependent changes in glutamic acid decarboxylase and parvalbumin in the adult rat brain. Neuroscience 287:78–92. 10.1016/j.neuroscience.2014.12.013 25528062

[b7] Benjamini Y, Hochberg Y (1995) Controlling the false discovery rate: a practical and powerful approach to multiple testing. J R Stat Soc 57:289–300. 10.1111/j.2517-6161.1995.tb02031.x

[b8] Bitanihirwe BK, Peleg-Raibstein D, Mouttet F, Feldon J, Meyer U (2010) Late prenatal immune activation in mice leads to behavioral and neurochemical abnormalities relevant to the negative symptoms of schizophrenia. Neuropsychopharmacology 35:2462–2478. 10.1038/npp.2010.129 20736993 PMC3055332

[b9] Borrell J, Vela JM, Arévalo-Martin A, Molina-Holgado E, Guaza C (2002) Prenatal immune challenge disrupts sensorimotor gating in adult rats. Implications for the etiopathogenesis of schizophrenia. Neuropsychopharmacology 26:204–215. 10.1016/S0893-133X(01)00360-8 11790516

[b10] Bronson SL, Ahlbrand R, Horn PS, Kern JR, Richtand NM (2011) Individual differences in maternal response to immune challenge predict offspring behavior: contribution of environmental factors. Behav Brain Res 220:55–64. 10.1016/j.bbr.2010.12.040 21255612 PMC3064713

[b11] Brown AS, Meyer U (2018) Maternal immune activation and neuropsychiatric illness: a translational research perspective. Am J Psychiatry 175:1073–1083. 10.1176/appi.ajp.2018.17121311 30220221 PMC6408273

[b12] Bussey T, Saksida L, Murray E (2005) The perceptual-mnemonic/feature conjunction model of perirhinal cortex function. Q J Exp Psychol Sect B 58:269–282. 10.1080/02724990544000004 16194969

[b13] Careaga M, Taylor SL, Chang C, Chiang A, Ku KM, Berman RF, Van de Water JA, Bauman MD (2018) Variability in PolyIC induced immune response: implications for preclinical maternal immune activation models. J Neuroimmunol 323:87–93. 10.1016/j.jneuroim.2018.06.014 30196839 PMC6782055

[b14] Clayton JA, Collins FS (2014) Policy: NIH to balance sex in cell and animal studies. Nature 509:282–283. 24834516 10.1038/509282a PMC5101948

[b15] Cloke JM, Nguyen R, Chung BYT, Wasserman DI, De Lisio S, Kim JC, Bailey CDC, Winters BD (2016) A novel multisensory integration task reveals robust deficits in rodent models of schizophrenia: converging evidence for remediation via nicotinic receptor stimulation of inhibitory transmission in the prefrontal cortex. J Neurosci 36:12570–12585. 10.1523/JNEUROSCI.1628-16.2016 27974613 PMC6705662

[b16] Coiro P, Pollak DD (2019) Sex and gender bias in the experimental neurosciences: the case of the maternal immune activation model. Transl Psychiatry 9:90. 10.1038/s41398-019-0423-8 30765690 PMC6375995

[b17] Cowell RA, Bussey TJ, Saksida LM (2010) Functional dissociations within the ventral object processing pathway: cognitive modules or a hierarchical continuum? J Cogn Neurosci 22:2460–2479. 10.1162/jocn.2009.21373 19929757

[b18] Davis EP, Pfaff D (2014) Sexually dimorphic responses to early adversity: implications for affective problems and autism spectrum disorder. Psychoneuroendocrinology 49:11–25. 10.1016/j.psyneuen.2014.06.014 25038479 PMC4165713

[b19] Dickerson DD, Wolff AR, Bilkey DK (2010) Abnormal long-range neural synchrony in a maternal immune activation animal model of schizophrenia. J Neurosci 30:12424–12431. 10.1523/JNEUROSCI.3046-10.2010 20844137 PMC6633437

[b20] Dickerson DD, Bilkey DK, Sandner G, Eyles DW (2013) Aberrant neural synchrony in the maternal immune activation model: using translatable measures to explore targeted interventions. Front Behav Neurosci 7:217. 24409130 10.3389/fnbeh.2013.00217 PMC3873515

[b21] Dickerson D, Overeem K, Wolff A, Williams J, Abraham W, Bilkey D (2014) Association of aberrant neural synchrony and altered GAD67 expression following exposure to maternal immune activation, a risk factor for schizophrenia. Transl Psychiatry 4:e418. 25072323 10.1038/tp.2014.64 PMC4119228

[b22] Du Y, Grace AA (2013) Peripubertal diazepam administration prevents the emergence of dopamine system hyperresponsivity in the MAM developmental disruption model of schizophrenia. Neuropsychopharmacology 38:1881–1888. 10.1038/npp.2013.101 23612434 PMC3746684

[b23] Du Y, Grace AA (2016a) Amygdala hyperactivity in MAM model of schizophrenia is normalized by peripubertal diazepam administration. Neuropsychopharmacology 41:2455–2462. 10.1038/npp.2016.42 27000940 PMC4987842

[b24] Du Y, Grace AA (2016b) Loss of parvalbumin in the hippocampus of MAM schizophrenia model rats is attenuated by peripubertal diazepam. Int J Neuropsychopharmacol 19:pyw065 10.1093/ijnp/pyw065 27432008 PMC5137280

[b26] Fortier MÈ, Joober R, Luheshi GN, Boksa P (2004) Maternal exposure to bacterial endotoxin during pregnancy enhances amphetamine-induced locomotion and startle responses in adult rat offspring. J Psychiatr Res 38:335–345. 10.1016/j.jpsychires.2003.10.001 15003440

[b27] Fortier ME, Luheshi GN, Boksa P (2007) Effects of prenatal infection on prepulse inhibition in the rat depend on the nature of the infectious agent and the stage of pregnancy. Behav Brain Res 181:270–277. 10.1016/j.bbr.2007.04.016 17553574

[b28] Gogos A, Van den Buuse M (2004) Estrogen and progesterone prevent disruption of prepulse inhibition by the serotonin-1A receptor agonist 8-hydroxy-2-dipropylaminotetralin. J Pharmacol Exp Ther 309:267–274. 10.1124/jpet.103.061432 14722325

[b29] Goldstein JM, Cherkerzian S, Seidman LJ, Donatelli J-A, Remington AG, Tsuang MT, Hornig M, Buka SL (2014) Prenatal maternal immune disruption and sex-dependent risk for psychoses. Psychol Med 44:3249–3261. 10.1017/S0033291714000683 25065485 PMC4477534

[b30] Gustafsson HC, Sullivan EL, Nousen EK, Sullivan CA, Huang E, Rincon M, Nigg JT, Loftis JM (2018) Maternal prenatal depression predicts infant negative affect via maternal inflammatory cytokine levels. Brain Behav Immun 73:470–481. 10.1016/j.bbi.2018.06.011 29920327 PMC6129422

[b31] Hadar R, Soto-Montenegro ML, Götz T, Wieske F, Sohr R, Desco M, Hamani C, Weiner I, Pascau J, Winter C (2015) Using a maternal immune stimulation model of schizophrenia to study behavioral and neurobiological alterations over the developmental course. Schizophr Res 166:238–247. 10.1016/j.schres.2015.05.010 26055633 PMC5233455

[b32] Henbid MT, Marks WN, Collins MJ, Cain SM, Snutch TP, Howland JG (2017) Sociability impairments in genetic absence epilepsy rats from Strasbourg: reversal by the T-type calcium channel antagonist Z944. Exp Neurol 296:16–22. 10.1016/j.expneurol.2017.06.022 28658605

[b33] Howland JG, Cazakoff BN, Zhang Y (2012) Altered object-in-place recognition memory, prepulse inhibition, and locomotor activity in the offspring of rats exposed to a viral mimetic during pregnancy. Neuroscience 201:184–198. 10.1016/j.neuroscience.2011.11.011 22119062 PMC4464820

[b34] Hubscher CH, Brooks DL, Johnson JR (2005) A quantitative method for assessing stages of the rat estrous cycle. Biotech Histochem 80:79–87. 10.1080/10520290500138422 16195173

[b35] Jacklin DL, Goel A, Clementino KJ, Hall AW, Talpos JC, Winters BD (2012) Severe cross-modal object recognition deficits in rats treated sub-chronically with NMDA receptor antagonists are reversed by systemic nicotine: implications for abnormal multisensory integration in schizophrenia. Neuropsychopharmacology 37:2322–2331. 10.1038/npp.2012.84 22669170 PMC3422496

[b36] Jacklin DL, Cloke JM, Potvin A, Garrett I, Winters BD (2016) The dynamic multisensory engram: neural circuitry underlying crossmodal object recognition in rats changes with the nature of object experience. J Neurosci 36:1273–1289. 10.1523/JNEUROSCI.3043-15.2016 26818515 PMC6604816

[b37] Jiang HY, Xu LL, Shao L, Xia RM, Yu ZH, Ling ZX, Yang F, Deng M, Ruan B (2016) Maternal infection during pregnancy and risk of autism spectrum disorders: a systematic review and meta-analysis. Brain Behav Immun 58:165–172. 10.1016/j.bbi.2016.06.005 27287966

[b38] Jovanovic T, Szilagyi S, Chakravorty S, Fiallos AM, Lewison BJ, Parwani A, Schwartz MP, Gonzenbach S, Rotrosen JP, Duncan EJ (2004) Menstrual cycle phase effects on prepulse inhibition of acoustic startle. Psychophysiology 41:401–406. 10.1111/1469-8986.2004.00166.x 15102125

[b39] Kentner AC, Bilbo SD, Brown AS, Hsiao EY, McAllister AK, Meyer U, Pearce BD, Pletnikov MV, Yolken RH, Bauman MD (2019) Maternal immune activation: reporting guidelines to improve the rigor, reproducibility, and transparency of the model. Neuropsychopharmacology 44:245–258. 10.1038/s41386-018-0185-7 30188509 PMC6300528

[b40] Klein J, Hadar R, Götz T, Männer A, Eberhardt C, baldassarri J, Schmidt TT, Kupsch A, Heinz A, Morgenstern R, Schneider M, Weiner I, Winter C (2013) Mapping brain regions in which deep brain stimulation affects schizophrenia-like behavior in two rat models of schizophrenia. Brain Stimul 6:490–499. 10.1016/j.brs.2012.09.004 23085443

[b41] Klein LC, Corwin EJ (2002) Seeing the unexpected: how sex differences in stress responses may provide a new perspective on the manifestation of psychiatric disorders. Curr Psychiatry Rep 4:441–448. 12441024 10.1007/s11920-002-0072-z

[b42] Kumari V (2011) Sex differences and hormonal influences in human sensorimotor gating: implications for schizophrenia, pp 141–154. Berlin; Heidelberg: Springer. 10.1007/7854_2010_117 21374020

[b43] Kumari V, Aasen I, Papadopoulos A, Bojang F, Poon L, Halari R, Cleare AJ (2008) A comparison of prepulse inhibition in pre- and postmenopausal women and age-matched men. Neuropsychopharmacology 33:2610–2618. 10.1038/sj.npp.1301670 18216776

[b44] Kuwagata M, Ogawa T, Shioda S, Nagata T (2009) Observation of fetal brain in a rat valproate-induced autism model: a developmental neurotoxicity study. Int J Dev Neurosci 27:399–405. 10.1016/j.ijdevneu.2009.01.006 19460635

[b45] Lazic SE (2013) Comment on “stress in puberty unmasks latent neuropathological consequences of prenatal immune activation in mice.” Science 340:811. 10.1126/science.1237793 23687029

[b46] Lee J, Green MF (2016) Social preference and glutamatergic dysfunction: underappreciated prerequisites for social dysfunction in schizophrenia. Trends Neurosci 39:587–596. 10.1016/j.tins.2016.06.005 27477199 PMC5951176

[b47] Lins BR, Pushie JM, Jones M, Howard DL, Howland JG, Hackett MJ (2016) Mapping alterations to the endogenous elemental distribution within the lateral ventricles and choroid plexus in brain disorders using x-ray fluorescence imaging. PLoS One 11:e0158152. 10.1371/journal.pone.0158152 27351594 PMC4924862

[b48] Lins BR, Hurtubise JL, Roebuck AJ, Marks WN, Zabder NK, Scott GA, Greba Q, Dawicki W, Zhang X, Rudulier CD, Gordon JR, Howland JG (2018) Prospective analysis of the effects of maternal immune activation on rat cytokines during pregnancy and behavior of the male offspring relevant to schizophrenia. eNeuro 5:ENEURO.0249-18.2018. 10.1523/ENEURO.0249-18.2018 PMC6140112 30225350

[b85] Lodge DJ, Grace AA (2001) Glutamatergic afferents from the hippocampus to the nucleus accumbens regulate activity of ventral tegmental area dopamine neurons. J Neurosci 21:4915–4922. 11425919 10.1523/JNEUROSCI.21-13-04915.2001 PMC6762358

[b49] Mac Giollabhui N, Breen EC, Murphy SK, Maxwell SD, Cohn BA, Krigbaum NY, Cirillo PM, Perez C, Alloy LB, Drabick DAG, Ellman LM (2019) Maternal inflammation during pregnancy and offspring psychiatric symptoms in childhood: timing and sex matter. J Psychiatr Res 111:96–103. 10.1016/j.jpsychires.2019.01.009 30690329 PMC6644717

[b50] Marks WN, Cain SM, Snutch TP, Howland JG (2016) The T-type calcium channel antagonist Z944 rescues impairments in crossmodal and visual recognition memory in genetic absence epilepsy rats from Strasbourg. Neurobiol Dis 94:106–115. 10.1016/j.nbd.2016.06.001 27282256

[b51] Marks WN, Parker ME, Zabder NK, Greba Q, Snutch TP, Howland JG (2018) T-type calcium channels in the orbitofrontal cortex mediate sensory integration as measured using a spontaneous oddity task in rats. Learn Mem 25:317–324. 10.1101/lm.047332.118 29907639 PMC6004062

[b52] Meyer U, Feldon J, Fatemi SH (2009) In-vivo rodent models for the experimental investigation of prenatal immune activation effects in neurodevelopmental brain disorders. Neurosci Biobehav Rev 33:1061–1079. 10.1016/j.neubiorev.2009.05.001 19442688

[b53] Missault S, Van Den Eynde K, Vanden Berghe W, Fransen E, Weeren A, Timmermans JP, Kumar-Singh S, Dedeurwaerdere S (2014) The risk for behavioural deficits is determined by the maternal immune response to prenatal immune challenge in a neurodevelopmental model. Brain Behav Immun 42:138–146. 10.1016/j.bbi.2014.06.013 24973728

[b54] Moriyama C, Galic MA, Mychasiuk R, Pittman QJ, Perrot TS, Currie RW, Esser MJ (2013) Prenatal transport stress, postnatal maternal behavior, and offspring sex differentially affect seizure susceptibility in young rats. Epilepsy Behav 29:19–27. 10.1016/j.yebeh.2013.06.017 23920381

[b55] Murray BG, Davies DA, Molder JJ, Howland JG (2017) Maternal immune activation during pregnancy in rats impairs working memory capacity of the offspring. Neurobiol Learn Mem 141:150–156. 10.1016/j.nlm.2017.04.005 28434949

[b56] Ogawa T, Kuwagata M, Hori Y, Shioda S (2007) Valproate-induced developmental neurotoxicity is affected by maternal conditions including shipping stress and environmental change during early pregnancy. Toxicol Lett 174:18–24. 10.1016/j.toxlet.2007.08.006 17900830

[b57] Ozawa K, Hashimoto K, Kishimoto T, Shimizu E, Ishikura H, Iyo M (2006) Immune activation during pregnancy in mice leads to dopaminergic hyperfunction and cognitive impairment in the offspring: a neurodevelopmental animal model of schizophrenia. Biol Psychiatry 59:546–554. 10.1016/j.biopsych.2005.07.031 16256957

[b58] Patel S, Masi A, Dale RC, Whitehouse AJO, Pokorski I, Alvares GA, Hickie IB, breen E, Guastella AJ (2018) Social impairments in autism spectrum disorder are related to maternal immune history profile. Mol Psychiatry 23:1794–1797. 10.1038/mp.2017.201 28993711

[b59] Patrich E, Piontkewitz Y, Peretz A, Weiner I, Attali B (2016) Maturation- and sex-sensitive depression of hippocampal excitatory transmission in a rat schizophrenia model. Brain Behav Immun 51:240–251. 10.1016/j.bbi.2015.08.021 26327125

[b60] Patterson PH (2011) Maternal infection and immune involvement in autism. Trends Mol Med 17:389–394. 10.1016/j.molmed.2011.03.001 21482187 PMC3135697

[b61] Paylor JW, Lins BR, Greba Q, Moen N, de Moraes RS, Howland JG, Winship IR (2016) Developmental disruption of perineuronal nets in the medial prefrontal cortex after maternal immune activation. Sci Rep 6:37580. 10.1038/srep37580 27876866 PMC5120325

[b62] Paylor JW, Wendlandt E, Freeman TS, Greba Q, Marks WN, Howland JG, Winship IR (2018) Impaired cognitive function after perineuronal net degradation in the medial prefrontal cortex. eNeuro 5:ENEURO.0253-18.2018. 10.1523/ENEURO.0253-18.2018 PMC6325561 30627657

[b63] Piontkewitz Y, Arad M, Weiner I (2011a) Abnormal trajectories of neurodevelopment and behavior following in utero insult in the rat. Biol Psychiatry 70:842–851. 10.1016/j.biopsych.2011.06.007 21816387

[b64] Piontkewitz Y, Arad M, Weiner I (2011b) Risperidone administered during asymptomatic period of adolescence prevents the emergence of brain structural pathology and behavioral abnormalities in an animal model of schizophrenia. Schizophr Bull 37:1257–1269. 10.1093/schbul/sbq040 20439320 PMC3196943

[b65] Piontkewitz Y, Arad M, Weiner I (2012) Tracing the development of psychosis and its prevention: what can be learned from animal models. Neuropharmacology 62:1273–1289. 10.1016/j.neuropharm.2011.04.019 21703648

[b66] Romero E, Ali C, Molina-Holgado E, Castellano B, Guaza C, Borrell J (2007) Neurobehavioral and immunological consequences of prenatal immune activation in rats. Influence of antipsychotics. Neuropsychopharmacology 32:1791–1804. 10.1038/sj.npp.1301292 17180123

[b67] Sangha S, Greba Q, Robinson PD, Ballendine SA, Howland JG (2014) Heightened fear in response to a safety cue and extinguished fear cue in a rat model of maternal immune activation. Front behav Neurosci 8:168. 10.3389/fnbeh.2014.00168 24847231 PMC4019856

[b68] Silverman JL, Yang M, Lord C, Crawley JN (2010) Behavioural phenotyping assays for mouse models of autism. Nat Rev Neurosci 11:490–502. 10.1038/nrn2851 20559336 PMC3087436

[b69] Smith SEP, Li J, Garbett K, Mirnics K, Patterson PH (2007) Maternal immune activation alters fetal brain development through interleukin-6. J Neurosci 27:10695–10702. 10.1523/JNEUROSCI.2178-07.2007 17913903 PMC2387067

[b70] Smolders S, Notter T, Smolders SMT, Rigo JM, Brône B (2018) Controversies and prospects about microglia in maternal immune activation models for neurodevelopmental disorders. Brain Behav Immun 73:51–65. 10.1016/j.bbi.2018.06.001 29870753

[b71] Swerdlow NR, Hartman PL, Auerbach PP (1997) Changes in sensorimotor inhibition across the menstrual cycle: implications for neuropsychiatric disorders. Biol Psychiatry 41:452–460. 10.1016/S0006-3223(96)00065-0 9034539

[b72] Van den Eynde K, Missault S, Fransen E, Raeymaekers L, Willems R, Drinkenburg W, Timmermans J-P, Kumar-Singh S, Dedeurwaerdere S (2014) Hypolocomotive behaviour associated with increased microglia in a prenatal immune activation model with relevance to schizophrenia. Behav Brain Res 258:179–186. 10.1016/j.bbr.2013.10.005 24129217

[b73] Vorhees CV, Graham DL, Braun AA, Schaefer TL, Skelton MR, Richtand NM, Williams MT (2015) Prenatal immune challenge in rats: effects of polyinosinic–polycytidylic acid on spatial learning, prepulse inhibition, conditioned fear, and responses to MK-801 and amphetamine. Neurotoxicol Teratol 47:54–65. 10.1016/j.ntt.2014.10.007 25450663 PMC4291316

[b74] Vorhees CV, Graham DL, Braun AA, Schaefer TL, Skelton MR, Richtand NM, Williams MT (2012) Prenatal immune challenge in rats: altered responses to dopaminergic and glutamatergic agents, prepulse inhibition of acoustic startle, and reduced route-based learning as a function of maternal body weight gain after prenatal exposure to Poly IC. Synapse 66:725–737. 10.1002/syn.21561 22473973 PMC3370146

[b75] Winters BD, Reid JM (2010) A distributed cortical representation underlies crossmodal object recognition in rats. J Neurosci 30:6253–6261. 10.1523/JNEUROSCI.6073-09.2010 20445051 PMC6632708

[b76] Wolff AR, Bilkey DK (2008) Immune activation during mid-gestation disrupts sensorimotor gating in rat offspring. Behav Brain Res 190:156–159. 10.1016/j.bbr.2008.02.021 18367260

[b77] Wolff AR, Bilkey DK (2010) The maternal immune activation (MIA) model of schizophrenia produces pre-pulse inhibition (PPI) deficits in both juvenile and adult rats but these effects are not associated with maternal weight loss. Behav Brain Res 213:323–327. 10.1016/j.bbr.2010.05.008 20471999

[b78] Wolff AR, Cheyne KR, Bilkey DK (2011) Behavioural deficits associated with maternal immune activation in the rat model of schizophrenia. Behav Brain Res 225:382–387. 10.1016/j.bbr.2011.07.033 21816179

[b79] Zhang Y, Cazakoff BN, Thai CA, Howland JG (2012) Prenatal exposure to a viral mimetic alters behavioural flexibility in male, but not female, rats. Neuropharmacology 62:1299–1307. 10.1016/j.neuropharm.2011.02.022 21376064 PMC4457519

[b80] Zhao YY, Li JT, Wang XD, Li YH, Huang RH, Su YA, Si TM (2013) Neonatal MK-801 treatment differentially alters the effect of adolescent or adult MK-801 challenge on locomotion and PPI in male and female rats. J Psychopharmacol 27:845–853. 10.1177/0269881113497613 23863926

[b81] Zorrilla EP (1997) Multiparous species present problems (and possibilities) to developmentalists. Dev Psychobiol 30:141–150. 9068968 10.1002/(sici)1098-2302(199703)30:2<141::aid-dev5>3.0.co;2-q

[b82] Zuckerman L, Weiner I (2003) Post-pubertal emergence of disrupted latent inhibition following prenatal immune activation. Psychopharmacology (Berl) 169:308–313. 10.1007/s00213-003-1461-7 12748757

[b83] Zuckerman L, Weiner I (2005) Maternal immune activation leads to behavioral and pharmacological changes in the adult offspring. J Psychiatr Res 39:311–323. 10.1016/j.jpsychires.2004.08.008 15725430

[b84] Zuckerman L, Rehavi M, Nachman R, Weiner I (2003) Immune activation during pregnancy in rats leads to a postpubertal emergence of disrupted latent inhibition, dopaminergic hyperfunction, and altered limbic morphology in the offspring: a novel neurodevelopmental model of schizophrenia. Neuropsychopharmacology 28:1778–1789. 10.1038/sj.npp.1300248 12865897

